# Plasmid-mediated virulence in *Chlamydia*

**DOI:** 10.3389/fcimb.2023.1251135

**Published:** 2023-08-17

**Authors:** Breanna J. Turman, Toni Darville, Catherine M. O'Connell

**Affiliations:** ^1^ Department of Microbiology and Immunology, University of North Carolina, Chapel Hill, NC, United States; ^2^ Department of Pediatrics, University of North Carolina, Chapel Hill, NC, United States

**Keywords:** *Chlamydia*, intracellular bacteria, bacterial pathogenesis, virulence plasmid, host pathogen interactions, virulence mechanisms

## Abstract

*Chlamydia trachomatis* infection of ocular conjunctiva can lead to blindness, while infection of the female genital tract can lead to chronic pelvic pain, ectopic pregnancy, and/or infertility. Conjunctival and fallopian tube inflammation and the resulting disease sequelae are attributed to immune responses induced by chlamydial infection at these mucosal sites. The conserved chlamydial plasmid has been implicated in enhancing infection, via improved host cell entry and exit, and accelerating innate inflammatory responses that lead to tissue damage. The chlamydial plasmid encodes eight open reading frames, three of which have been associated with virulence: a secreted protein, Pgp3, and putative transcriptional regulators, Pgp4 and Pgp5. Although Pgp3 is an important plasmid-encoded virulence factor, recent studies suggest that chlamydial plasmid-mediated virulence extends beyond the expression of Pgp3. In this review, we discuss studies of genital, ocular, and gastrointestinal infection with *C. trachomatis* or *C. muridarum* that shed light on the role of the plasmid in disease development, and the potential for tissue and species-specific differences in plasmid-mediated pathogenesis. We also review evidence that plasmid-associated inflammation can be independent of bacterial burden. The functions of each of the plasmid-encoded proteins and potential molecular mechanisms for their role(s) in chlamydial virulence are discussed. Although the understanding of plasmid-associated virulence has expanded within the last decade, many questions related to how and to what extent the plasmid influences chlamydial infectivity and inflammation remain unknown, particularly with respect to human infections. Elucidating the answers to these questions could improve our understanding of how chlamydia augment infection and inflammation to cause disease.

## 1 Introduction

In humans, *Chlamydia trachomatis* infects mucosal sites, including ocular conjunctiva, the genital and gastrointestinal (GI) tracts and the respiratory tract of newborns. Infection of the eye leads to conjunctival scarring and entropion (inward turning of eyelids and lashes) resulting in corneal scarring or trichiasis, and eventual blindness. The tissue tropism of *C. trachomatis* infection can be partially defined by chlamydial serovar. *C. trachomatis* serovars A-C are the leading cause of infectious blindness worldwide (WHO, 2022). It is estimated that approximately 1.9 million people have suffered blindness or visual impairment from trachoma (WHO, 2022). Children who live in endemic areas become infected multiple times, which, in genetically susceptible individuals, can lead to the slow development of conjunctival scarring resulting in blindness in adulthood (WHO, 2022).

*C. trachomatis* infection of the reproductive tract is caused by serovars D-K and L1-L3 and is the most common bacterial sexually transmitted infection (STI) in the United States and globally. In 2020 there were 1.6 million cases of chlamydial STI in the U.S.; representing a 20% increase in cases since 2010 (National Center for HIV, 2021). In women, chlamydial infection begins at the endocervix, however, the bacteria can ascend the reproductive tract to infect the uterus and fallopian tubes. Ascension to the upper reproductive tract can lead to pelvic inflammatory disease (PID), and long-term complications of ectopic pregnancy, chronic pelvic pain, and/or infertility (Centers for Disease Control and Prevention, 2022) resulting from irreversible tissue damage caused by an overly robust host immune response.

More recently, *C. trachomatis* has been isolated from the rectum of men and women (Schachter et al., 1986; Geisler et al., 2002; Kent et al., 2005; Peters et al., 2011; Javanbakht et al., 2012; Gratrix et al., 2014). Studies suggest that approximately 4 to 8% of men (Geisler et al., 2002; Kent et al., 2005) and up to 14.6% of women (Peters et al., 2011; Javanbakht et al., 2012; Gratrix et al., 2014) have anorectal infection. Among women, rectal infection is more common among, but not exclusive to, individuals with coincident genital tract infection (Peters et al., 2011; Javanbakht et al., 2012). Currently, there is no evidence that chlamydial anorectal infection with serovars D-K leads to localized inflammation or disease. Rather *Chlamydia* may act as a symbiont with GI flora. However, infants presumed to be colonized rectally at birth as a consequence of vertical transmission from their genitally infected mothers resolved spontaneously within one year (Schachter et al., 1986) suggesting *C. trachomatis* clears from GI tract tissue.

Much of our knowledge of chlamydial pathogenesis in the eye, genital tract, and GI tract comes from studies of naturally infected animals such as guinea pigs and pigs. Non-human primates provide a model for human *C. trachomatis* ocular and genital tract infection and disease (Patton et al., 1983; Kari et al., 2008). Genital tract infection can be modeled in mice using human *C. trachomatis* strains or *C. muridarum*, a species-specific pathogen of the murine respiratory tract. Vaginal inoculation of female mice with *C. muridarum* (formerly known as Mouse Pneumonitis Agent) causes an acute, self-limiting infection. Like infection in women, *C. muridarum* ascends from the endocervix to the uterine horns and oviducts, eliciting host inflammatory responses that lead to pathology in the form of uterine hydrometra and post-obstructive oviduct dilatation or hydrosalpinx, making this genital tract model excellent for studying disease pathogenesis.

Animal studies indicate that the conserved chlamydial plasmid contributes to chlamydial virulence and disease following infection. In this review, we begin with an outline of the chlamydial developmental cycle and a brief overview of the immune response to chlamydial infection. We discuss the multi-faceted roles of the chlamydial plasmid in chlamydial virulence during ocular, reproductive tract, and gastrointestinal infection. Lastly, we provide an in-depth review of three plasmid-encoded virulence proteins: Pgp3, Pgp4, and Pgp5, and discuss the proposed molecular mechanisms for their roles in plasmid-associated virulence during chlamydial infection.

## 2 *Chlamydia* are obligate intracellular bacteria

*Chlamydia* spp. are Gram-negative obligate intracellular bacteria with a unique bi-phasic developmental cycle. Chlamydiae exist as two developmental forms: the infectious and metabolically inert elementary body (EB) and the replicative, metabolically active, reticulate body (RB) (Elwell et al., 2016). To initiate infection of a host epithelial cell, a chlamydial EB binds to the surface using bacterial proteins and host receptors. Chlamydial adhesion to host cells is not well understood but studies suggest that multiple bacterial proteins are involved (Su et al., 1988; Su et al., 1990; Fadel and Eley, 2007; Moelleken and Hegemann, 2007; Mölleken et al., 2010; Fechtner et al., 2013; Mölleken et al., 2013; Becker and Hegemann, 2014; Paes et al., 2018). The surface-exposed, envelope protein OmcB binds glycosaminoglycans on the host cell surface (Fadel and Eley, 2007; Moelleken and Hegemann, 2007; Fadel and Eley, 2008; Fechtner et al., 2013; Liang et al., 2021), while the chlamydial major outer membrane protein (MOMP) was shown to potentially bind heparan sulfate receptors on the host cell (Su et al., 1996). Binding of the epithelial growth factor receptor (EGFR) by chlamydial Ctad1(Stallmann and Hegemann, 2016) and engagement of β1-integrin by bacterial polymorphic membrane proteins (Mölleken et al., 2013; Luczak et al., 2016; Li et al., 2022) have also been proposed as adhesion mechanisms. It is likely that multiple mechanisms are combined and utilized by chlamydial EBs for adhesion, given the importance of binding and entry to its intracellular developmental cycle.

Upon attachment to the host cell, chlamydial EBs secrete pre-packaged bacterial effectors such as Tarp (Clifton et al., 2004; Chen et al., 2014; Parrett et al., 2016; Ghosh et al., 2020), TepP (Chen et al., 2014), CT694 (Hower et al., 2009), CT695 (Mueller and Fields, 2015), and TmeA (Mckuen et al., 2017) into the host cell cytosol using their Type III Secretion System (T3SS). These effectors promote uptake of the bacterium into the host cell via rearrangements to the host cell cytoskeleton. Once inside the cell, the EB differentiates into an RB and replicates in a membrane bound vacuole called the inclusion. Throughout the developmental cycle, chlamydiae secrete many bacterial effectors into the host cell cytosol to modulate host cell processes including vesicular trafficking (Moore et al., 2008; Capmany and Damiani, 2010; Sixt et al., 2017; Weber et al., 2017; Faris et al., 2019; Pais et al., 2019; Auer et al., 2020), pathogen sensing and inflammatory signaling (Carpenter et al., 2017; Sixt et al., 2017), and cell death (Fan et al., 1998; Fischer et al., 2004; Waguia Kontchou et al., 2016; Fischer et al., 2017; Sixt et al., 2017). Near the end of the developmental cycle, RBs convert back into EBs in an asynchronous manner. Newly made EBs are then released from the infected cell via cell lysis or a process called extrusion (Hybiske and Stephens, 2007), where the inclusion or a portion of the inclusion is extruded from the infected cell leaving the cell and inclusion membrane intact. Released EBs reinitiate the developmental cycle by binding adjacent host cells.

## 3 The immune response to *chlamydia* infection

*Chlamydia*-induced tissue damage is the result of the host immune response to infection. Infected epithelial cells recognize chlamydiae via pattern recognition receptors (PRRs) on and within the host cell. To date, chlamydiae have been demonstrated to activate cGAS (Zhang et al., 2014), STING (Prantner et al., 2010; Barker et al., 2013), NOD1 (Welter-Stahl et al., 2006; Buchholz and Stephens, 2008), Caspase-11 (Finethy et al., 2015; Allen et al., 2019), TLR2 (Darville et al., 2003; O’Connell et al., 2006; O’Connell et al., 2011), and TLR4 (Ingalls et al., 1995; Sasu et al., 2001), with Caspase-11 (Allen et al., 2019) and TLR2 (Darville et al., 2003) contributing to immunopathology (Allen et al., 2019).

Chlamydiae activate the caspase-11 inflammasome in mice (Finethy et al., 2015; Allen et al., 2019) likely via lipopolysaccharide (LPS) (Hagar et al., 2013). Humans express caspase-4 and -5 rather than caspase-11 (Shi et al., 2015; Matikainen et al., 2020) rendering it challenging to make direct comparisons between the mouse model and human disease. However, their activation leads to the production of IL-1β and IL-1α (Viganò et al., 2015; Matikainen et al., 2020). IL-1α directly damages the epithelial layer, revealed by the extensive tissue destruction that can be observed in *C. trachomatis*-infected human fallopian tube explants, and blocked by addition of IL-1 receptor antagonist (IL1RA) (Hvid et al., 2007). IL-1α is more important than IL-1α for induction of oviduct pathology following *C. muridarum* genital tract infection (Gyorke et al., 2020). These data suggest that production of IL-1α in humans after caspase -4 and -5 activation could contribute to disease.

Chlamydiae also activate TLR2, a toll-like receptor expressed on plasma and endosomal membranes of multiple cell types including epithelial and immune cells. Bacterial lipoproteins and polysaccharides activate TLR2 in other infection models (reviewed in (De Oliviera Nascimento et al., 2012)), but the chlamydial TLR2 ligand remains unidentified. TLR2 and its adapter protein MYD88 localize to the chlamydial inclusion (O’Connell et al., 2006) during infection suggesting the ligand is sampled within the inclusion during infection. Once activated, TLR2 recruits MYD88 and initiates a signaling cascade to ultimately activate transcription factors NFκB and/or AP-1 via the activation of mitogen activated protein kinases (MAPK) such as p38 (De Oliviera Nascimento et al., 2012). Activation of NFκB and/or AP-1 leads to the production and secretion of cytokines such as TNF-α, IL-6, and IL-8 (De Oliviera Nascimento et al., 2012). Production of TNF-α could partially account for the role of TLR2 in pathology because TNF-α production and signaling has been associated with more severe disease following *Chlamydia* infection in humans (Öhman et al., 2009) and in mice (Murthy et al., 2011; Kamalakaran et al., 2013). Activation of TLR2 by *Chlamydia* also leads to the production of chemokines that recruit immune cells, including neutrophils to the infected genital tract (Darville et al., 2003; O’Connell et al., 2007; Frazer et al., 2011).

Studies have established that neutrophils are the primary cells driving tissue damage during infection (O’Connell et al., 2007; Lee et al., 2010; Frazer et al., 2011; Lacy et al., 2011; Lei et al., 2014; Lijek et al., 2018). In mice, intensive recruitment, and activation of neutrophils in oviducts results in scarring and subsequent post-obstructive dilatation or hydrosalpinx (O’Connell et al., 2007; Lee et al., 2010; Frazer et al., 2011; Lei et al., 2014). Neutrophil infiltration of conjunctiva has also been associated with increased pathology and scarring with ocular infection (Lacy et al., 2011). Neutrophil-driven pathology is likely the result of multiple mechanisms including physical dislodging of cells from the epithelium (Rank et al., 2008; Rank et al., 2011) and the production of tissue-damaging proteins such as matrix metalloproteinase-9 (MMP-9) (El-Asrar et al., 2000; Ramsey et al., 2005; Natividad et al., 2006; Imtiaz et al., 2007). Not only are neutrophils strongly associated with tissue damage, their contribution to chlamydial clearance is minor as shown by antibody-mediated depletion of neutrophils during murine genital tract infection, which resulted in no alteration of chlamydial shedding but reduced oviduct pathology (Lijek et al., 2018). The chlamydial serine protease, CPAF, paralyzes neutrophils, preventing their production of neutrophil extracellular traps (NETS) and reactive oxygen species important for bacterial killing (Rajeeve et al., 2018). An adaptive immune response characterized by cooperation between antibodies, CD4 ^+^ T cells, and phagocytes is required for bacterial clearance (Morrison et al., 2000; Morrison and Morrison, 2001; Morrison and Morrison, 2005).

In contrast, chlamydial infection of the GI tract does not elicit a robust innate immune response (Igietseme et al., 2001; Yeruva et al., 2013). Immune cell infiltrates or histological changes are not observed in the GI tracts of mice orally infected with *C. muridarum* up to 240 days post-infection (Igietseme et al., 2001). Despite evidence against the existence of an innate immune response following gastric inoculation, mice with GI tract infection develop anti-chlamydial IgG and IgA responses, as well as T cell responses that peak between days 10-25 post-infection before returning to baseline levels (Yeruva et al., 2013). In humans, anti-chlamydial antibodies have also been detected in infants with rectal infection without signs of GI distress (Schachter et al., 1986). The specific mechanism(s) that differentiate the immune response to chlamydial infection in the GI tract from responses in the eye or reproductive tract are currently unknown but may be related to overall dampening of immune responses in the GI tract by microbiota.

## 4 Loss of the chlamydial plasmid is pleiotropic

Many *Chlamydia* spp. including *C. trachomatis* and *C. muridarum*, contain a conserved 7.5 kb plasmid. The chlamydial plasmid is nearly ubiquitous among *C. trachomatis* clinical isolates and only a handful of plasmid-less isolates have been described (Peterson et al., 1990; Farencena et al., 1997; Matsumoto et al., 1998; Stothard et al., 1998) suggesting the chlamydial plasmid is important for bacterial fitness. Isolation of spontaneous plasmid-deficient *C. trachomatis* strains (Matsumoto et al., 1998) and novobiocin-mediated curing of the chlamydial plasmid from *C. muridarum* (O’Connell and Nicks, 2006) provided some of the first clear evidence that plasmid loss in *Chlamydia* is pleiotropic. Plasmid-deficient *Chlamydia* exhibit an infectivity defect, elicit reduced levels of inflammation and pathology, and do not accumulate glycogen in their inclusions. The plasmid-associated infectivity defect has been described in cell culture (O’Connell and Nicks, 2006; O’Connell et al., 2011; Russell et al., 2011), in a mouse model of genital tract infection (O’Connell et al., 2007; Russell et al., 2011; Lei et al., 2014; Sigar et al., 2014) and in gastrointestinal infection (Shao et al., 2017; Ma et al., 2020). Similarly, ocular infection with plasmid-deficient *C. trachomatis* in cynomolgus macaques resulted in accelerated clearance of the mutant when compared to wild-type (Kari et al., 2011).

The mechanism(s) underlying the plasmid-associated infectivity defect remain unknown and the subject of controversy (**Table 1**). O’Connell and Nicks hypothesized that the plasmid-associated infectivity defect they detected using a cell-culture based plaque assay resulted in reduced binding and entry (O’Connell and Nicks, 2006) because it could be overcome by centrifugation. Subsequently, this group isolated spontaneous suppressors of this phenotype (O’Connell et al., 2007; Russell et al., 2011; Lei et al., 2014; Sigar et al., 2014) and demonstrated that one of these mutants, strain CM3.1, was not disadvantaged in competition against its plasmid-containing ancestor during sequential rounds of synchronous cell passage (Russell et al., 2011) and ascended efficiently to the upper genital tract of mice (O’Connell et al., 2007; Russell et al., 2011; Lei et al., 2014; Sigar et al., 2014). Whole genome sequencing of the mutant identified a single nucleotide polymorphism predicted to prematurely terminate expression of TC_236, a protein of unknown function (Russell et al., 2011). However, their study also isolated suppressor variants that were wild type at this locus, so its biological significance remains unclear. Skilton et al. described subtle differences in the overall growth profile of plasmid-bearing and plasmid-deficient *C. muridarum* (Skilton et al., 2018) and proposed these could contribute to reduced infection. Recently, earlier onset of RB-to-EB conversion within the developmental cycle has been described for a *C. muridarum* strain carrying a deletion in the plasmid-encoded regulator Pgp4 that may explain these observations (Zhang et al., 2020; Grieshaber et al., 2022) but no differences in overall EB yield were reported. One study has generated data suggesting that carriage of the plasmid promotes chlamydial exit from infected cells in culture (Yang et al., 2015), while infection studies in mice have investigated if reduced infectivity associated with plasmid loss reflects an inability to neutralize the anti-chlamydial cathelicidin-related antimicrobial peptide (CRAMP) (Hou et al., 2015; Yang et al., 2020).

**Table 1:** Putative mechanisms for plasmid-dependent effects on infectivity and inflammation.

	Mechanism	Reference(s)
**Infectivity**	Increased binding and entry	(O’Connell and Nicks, 2006)
	Improved growth/RB to EB conversion	(Skilton et al., 2018; Zhang et al., 2020; Grieshaber et al., 2022)
	Neutralization of antimicrobial peptide LL-37/CRAMP	(Hou et al., 2015; Yang et al., 2020)
	Enhanced exit	(Yang et al., 2015)
	Efficient packaging and secretion of outer membrane vesicles (indirect)	(Turman et al., 2023)
	Acid tolerance	(Zhang et al., 2019)
**Inflammation**	Control of TLR2 activation	(O’Connell et al., 2007; Frazer et al., 2011; He et al., 2011; O’Connell et al., 2011; Zhou et al., 2013)
	Inhibition of apoptosis	(Zou et al., 2018; Zou et al., 2019; Luo et al., 2020; Shu et al., 2023)
	Modulation of antimicrobial peptide LL-37	(Hou et al., 2019; Yang et al., 2020)

Genital tract infection of mice with wild-type *C. muridarum* also leads to colonization of the GI tract, primarily in the cecum and large intestine (Yeruva et al., 2013). Plasmid-deficient *C. muridarum* appear less capable of colonizing mice infected intravaginally, a deficit that is most pronounced during early infection. (Shao et al., 2017; Ma et al., 2020). Mice infected intragastrically with plasmid-deficient *C. muridarum* exhibit reduced rectal shedding and decreased infectious progeny in GI tract tissue (Shao et al., 2017; Ma et al., 2020), most pronounced in the small intestine (Ma et al., 2020). Significantly, *C. muridarum* disseminates systemically in mice, providing an additional, hematogenous, route for spread from the genital tract to the GI tract. In contrast, *C. trachomatis* genital serovars remain mucosally restricted in humans.

Plasmid-associated inflammation is independent of infectivity. Phagocytic murine bone marrow-derived dendritic cells incubated with live or UV-inactivated plasmid-deficient *C. muridarum* and *C. trachomatis* produce less TNF-α and IL-6 (O’Connell et al., 2011) than cells incubated with wild-type *C. muridarum* and *C. trachomatis*. Similarly, human epithelial cells infected with plasmid-less *C. trachomatis*via centrifugation secrete less GM-CSF, IL-6 and IL-8 (Lehr et al., 2018) than wild-type infected cells. Importantly, the plasmid-deficient strain of *C. muridarum*, CM3.1, which carries a suppressor mutation that restores infectivity, still elicits reduced inflammation in cell culture and significantly reduced genital tract pathology in infected mice (O’Connell et al., 2007; Frazer et al., 2011). The lower levels of TLR2-dependent cytokine secretion by mice infected with CM3.1 correlates with significantly less neutrophil recruitment to the oviducts, but recruitment of adaptive CD4^+^ T cells remains unimpaired and bacteria are cleared from the genital tract without delay (O’Connell et al., 2007; Frazer et al., 2011). Reduced cytokine secretion and pathology observed in mice infected with plasmid-deficient *C. muridarum* and sacrificed early post-infection resolution (O’Connell et al., 2007; Lei et al., 2014) has been attributed to a failure to activate TLR2 (O’Connell et al., 2007; O’Connell et al., 2011). Although the chlamydial TLR2-ligand(s) remains unknown, activation of HEK-TLR2-reporter cells by live and UV-inactivated wild-type *C. muridarum* and *C. trachomatis* (O’Connell et al., 2011), but not by their isogenic plasmid-deficient derivatives, provides direct evidence for plasmid-dependent activation of TLR2, independent of chlamydial growth and replication.

Wild-type *C. trachomatis* infection does not elicit gross oviduct pathology in infected mice (Gondek et al., 2012; Rajeeve et al., 2018) impeding investigation of the plasmid’s role in hydrosalpinx formation during murine infection. However, genital tract infection of macaques multiply inoculated/challenged with wild-type *C. trachomatis* serovar D and an isogenic plasmid-deficient strain revealed no evidence of plasmid-associated differences in bacterial burden or pathology (Qu et al., 2015; Patton et al., 2018). Rather, genetic diversity associated with differential immune responses between individual animals led to divergent infection outcomes. Monkeys with higher CD4^+^ T cell proliferation in response to purified *C. trachomatis* serovar D EBs, were protected from challenge infection and pathology, while enhanced infection and detection of tissue pathology were observed in monkeys with lower CD4^+^ T cell responses and increased antibody titers. These differences failed to associate with presence or absence of the plasmid (Qu et al., 2015) and contrast with a clear association between plasmid carriage and extended conjunctival infection demonstrated by the studies of Kari et al., (Kari et al., 2008). Genetic studies suggest that the chlamydial plasmid has co-evolved with its strain/serovar (Seth-Smith et al., 2009; Jelocnik et al., 2016; Versteeg et al., 2018) so whether these differences reflect altered roles for plasmid-encoded or regulated loci between ocular and genital strains or altered hierarchies of host innate inflammatory responses at these different mucosal sites remains to be investigated.

## 5 Plasmid loci Pgp3, Pgp4, and Pgp5 are associated with virulence

The chlamydial plasmid encodes eight open reading frames (*pgp1*-*pgp8*) and two non-coding RNAs (Albrecht et al., 2010; Ferreira et al., 2013). Technological advances in genetic manipulation and transformation of *Chlamydia* have enabled studies of the role(s) played by individual plasmid-encoded genes and how they contribute to the phenotypes associated with plasmid-deficiency (Gong et al., 2013; Song et al., 2013; Liu et al., 2014a; Liu et al., 2014b; Ramsey et al., 2014; Yang et al., 2015; Shao et al., 2018; Yang et al., 2020; Turman et al., 2023). The open reading frames *pgp1*, -*2*, -*6*, and-*8* encode proteins important for plasmid replication or maintenance (Song et al., 2013). Pgp4 is a putative transcriptional regulator that is required for expression of plasmid-encoded *pgp3* and chromosomal loci including *glgA*, the gene that encodes glycogen synthase (Carlson et al., 2008; Song et al., 2013). Pgp3 is a trimeric protein (Chen et al., 2010; Galaleldeen et al., 2013; Khurshid et al., 2018) that is released into the host cytosol during infection. Pgp5 encodes a putative transcriptional regulator (Liu et al., 2014a) that has been proposed to negatively regulate plasmid-regulated chromosomal loci (Huang et al., 2015).

Of the eight open reading frames on the chlamydial plasmid, *pgp3*, *pgp4*, and *pgp5* have been associated with virulence and disease. Double deletion of *pgp3*- and *pgp4*- from a relatively low passaged *C. trachomatis* E clinical isolate resulted in an infectivity defect in cell culture and in the murine genital tract model (Turman et al., 2023). Pgp3- and Pgp4- deficient *C. trachomatis* serovar D is rapidly cleared from the murine genital tract when compared to wild-type (Yang et al., 2020). Deletion of either *pgp3* or *pgp4* results in an infectivity defect in *C. muridarum* and *C. trachomatis* during infection of the murine genital tract (Liu et al., 2014b; Ramsey et al., 2014) and reduced spread of *C. muridarum* from the genital tract to the gut (Shao et al., 2018). Intravaginal infection with Pgp3-deficient or Pgp4-deficient *C. muridarum* results in reduced oviduct pathology in mice (Liu et al., 2014b). Deletion of *pgp5* from *C. muridarum* was associated with reduced ascension to the oviduct and reduction in hydrosalpinx frequency when compared to infection with wild-type (Huang et al., 2015). These results indicate that Pgp3, Pgp4, and Pgp5 are important plasmid-encoded virulence factors for *Chlamydia*.

## 6 Pgp3 is involved in chlamydial infectivity and inflammation during infection

Pgp3 is a unique trimeric protein that exhibits minimal protein homology to other known proteins at the amino acid sequence or structural level (Galaleldeen et al., 2013; Khurshid et al., 2018). Pgp3 is well conserved among *Chlamydia* spp. with ≥ 96% homology between *C. trachomatis* serovars and 82% homology between *C. trachomatis* and *C. muridarum* (Li et al., 2008) and is immunodominant during *Chlamydia* infection with up to 70% of infected people developing anti-Pgp3 antibody (Horner et al., 2016; Woodhall et al., 2017; Blomquist et al., 2018; Anyalechi et al., 2021). The immunodominance of Pgp3 likely stems from its secretion during infection. Pgp3 is secreted into the infected cell cytosol late during the infection cycle near the end of replication and the beginning of RB-EB conversion (Li et al., 2008; Lei et al., 2021). Multiple molecular functions for Pgp3 during infection have been proposed. It has been postulated that secreted Pgp3 is released from infected epithelial cells upon cell lysis and binds to the antimicrobial peptide LL-37 in humans (Hou et al., 2015; Hou et al., 2019) or CRAMP in mice (Yang et al., 2020) in the extracellular space to counter their anti-chlamydial functions. Binding to antimicrobial peptides has also been proposed to contribute to modulation of the immune response by Pgp3. Hou et al. showed that Pgp3 binding to LL-37 decreased neutrophil chemotaxis, but induced cytokine secretion from neutrophils and macrophages (Hou et al., 2019). This mechanism could contribute to the role of Pgp3 in infectivity and inflammation during genital tract infection, but it does not explain the reduced infectivity and cytokine secretion when plasmid-deficient strains are cultured in cells that do not produce antimicrobial peptides (O’Connell and Nicks, 2006; O’Connell et al., 2011). These observations suggest that other plasmid-encoded or -regulated loci may be involved in plasmid-associated infectivity or that Pgp3 has multiple roles in chlamydial infectivity and inflammation. The mechanism by which Pgp3 is secreted is unclear but appears to be conserved between *C. trachomatis* serovars and *C. muridarum* (Li et al., 2008). The need for Pgp3 to cross the bacterial membrane as well as the inclusion membrane to localize in cytosol led to speculation that it was secreted via the T3SS. However, Pgp3 secretion is insensitive to treatment with the T3SS inhibitor, Compound 1 (Lei et al., 2021).

**Figure 1:**
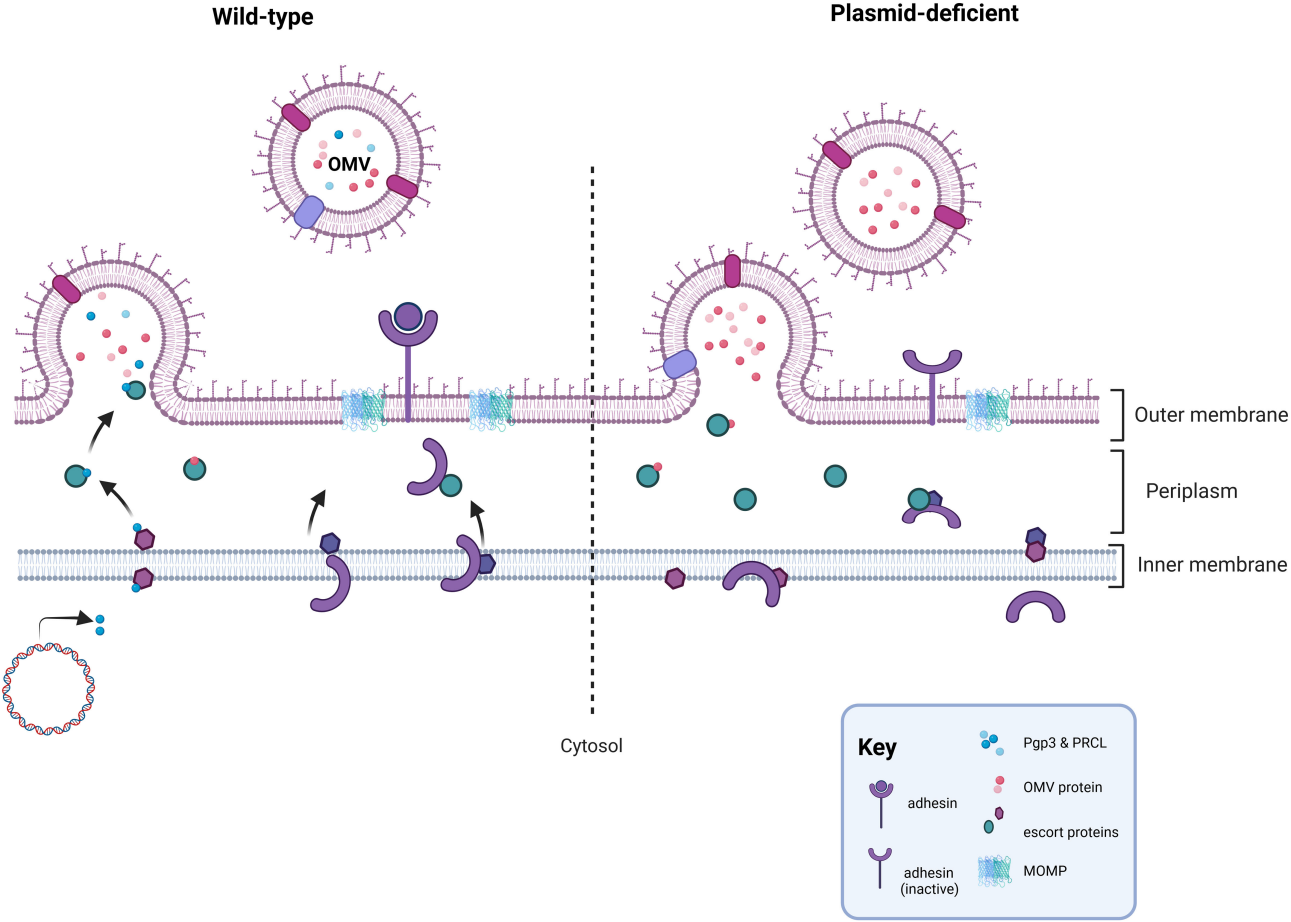
Proposed mechanism for the role of the plasmid in infectivity. In wild-type *Chlamydia*, the secretion pathways needed for OMV-mediated secretion of Pgp3 or Pgp4-regulated, chromosomally encoded secreted proteins functions properly and facilitates events at the chlamydial membrane important for the formation of a functional EB, such as placement of an adhesin in the outer membrane. In the absence of the plasmid, or in strains lacking Pgp3 or Pgp4, the OMV secretion pathway is negatively impacted leading to off target effects at the chlamydial membrane coinciding with RB-EB transition. Disruption of the OMV secretion pathway could impact the proper placement, folding, or cross-linking of a chlamydial adhesin in the outer membrane. Figure was created using BioRender.com.

Studies examining the contents of outer membrane vesicles (OMVs) have identified Pgp3 as a potential cargo protein providing an alternative mechanism for Pgp3 release (Frohlich et al., 2012; Frohlich et al., 2014). Immunofluorescent staining of Pgp3 and other secreted Pgp4-regulated chromosomal studies supported a model in which plasmid-regulated secreted effectors may be packaged and secreted together via OMVs in a Pgp4-dependent manner (Lei et al., 2021). Pgp3 secretion is not observed in the absence of Pgp4 (Lei et al., 2021; Turman et al., 2023) and the importance of actively secreting Pgp3 to chlamydial infectivity in cell culture was recently demonstrated when we observed that a strain independently expressing Pgp3, achieved by placing *pgp3* under the control of an anhydrotetracycline inducible promoter, in the absence of Pgp4 was insufficient to render it competitive with a wild type strain (Turman et al., 2023). Based upon this apparent need for Pgp3 cytosolic secretion, we recently proposed an indirect role for Pgp3 in plasmid-associated infectivity (**Figure 1**). We speculated that disruption of a plasmid-associated secretion system by deletion of the entire plasmid, *pgp3*, or *pgp4*, impedes events at the membrane that contribute to the generation of optimally infectious EB. Evidence supports potential changes to OmcB in the membrane of plasmid-deficient *C. muridarum* (Turman et al., 2023). An indirect mechanism for plasmid-associated infectivity does not conflict with other proposed models for the role of Pgp3 in infectivity. The isolation of plasmid-deficient *C. muridarum* strains with restored infectivity via multiple suppressor mutations further supports an indirect role for the plasmid in chlamydial infectivity (O’Connell et al., 2007). However, additional studies are needed to provide more rigorous evidence for this model.

Pgp3 was also suggested to help *Chlamydia* survive in acidic environments such as the stomach and genital tract following a study that showed Pgp3 was associated with increased survival in gastric acid and lactic acid in cell culture (Zhang et al., 2019). This model is supported by observations that deletion of Pgp3 in *C. muridarum* results in reduced colonization of the GI tract compared to wild-type, when mice were inoculated intragastrically (Shao et al., 2018). In addition to the ability to survive stomach acid, Pgp3-dependent acid tolerance was provided as a potential explanation for reduced recovery of Pgp3-deficient *C. muridarum* from the lower genital tracts of mice (Zhang et al., 2019). However, previous studies showed that the *pgp3*-associated infectivity defect in *C. muridarum* is related to with reduced bacterial burdens in the upper genital tract, rather than the lower genital tract (Liu et al., 2014b). Despite questions surrounding the relevance of Pgp3-associated acid tolerance in genital tract infection, the observation that Pgp3-deficient *C. muridarum* are more sensitive to killing by gastric acid and lactic acid in cell culture supports our proposed model of *pgp3*-deficiency leading to changes in the outer membrane complex (Turman et al., 2023) because these changes could lead to increased sensitivity to acid. However, in the model we proposed, acid tolerance is unlikely to be the direct cause of the infectivity defect, but rather an off-target consequence of membrane or surface changes arising from plasmid-, *pgp3*-, and *pgp4* deficiency (Turman et al., 2023). Alternate roles for Pgp3 during infection have been proposed. The use of recombinant protein or ectopic expression in the absence of infection have made interpreting these studies in the context of chlamydial infection challenging. For example, recombinant Pgp3 fused to red fluorescent protein (RFP) induced TNF-α and MIP-2 secretion from stimulated mouse macrophages (Li et al., 2008) and was subsequently suggested to induce pro-inflammatory cytokine secretion from macrophages via activation of TLR2 in a p38 MAPK-dependent manner (Zhou et al., 2013; Cao et al., 2015). However, other studies showed no cytokine secretion was induced in human monocytes and macrophages by recombinant Pgp3 (Bas et al., 2008).

## 7 Pgp4 and Pgp5, putative regulators of chlamydial virulence

The role of Pgp4 in virulence has been attributed to its function as a putative transcriptional regulator. Pgp4 was identified as a putative transcriptional activator following the study by Song et al. where reduced transcription of *pgp3* and previously identified, plasmid-responsive chromosomal loci (PRCLs) (Carlson et al., 2008; O’Connell et al., 2011) was observed in Pgp4-deficient *C. trachomatis* (Song et al., 2013). Regulation of glycogen synthase, GlgA, by Pgp4 is thought to account for glycogen accumulation in the chlamydial inclusion (Carlson et al., 2008; O’Connell et al., 2011; Song et al., 2013). The absence of Pgp4 and consequent reduced *glgA* transcription in plasmid-deficient *C. trachomatis* leads to lack of glycogen accumulation (Song et al., 2013). In contrast, plasmid-deficient *C. muridarum* transcribe *glgA* independently of the plasmid, expressing enzymatically active glycogen synthase at levels comparable to wild type (Carlson et al., 2008; O’Connell et al., 2011; Song et al., 2013), but still fail to accumulate glycogen within their inclusions. Pgp4 has also been implicated in the differential exit observed between plasmid-deficient and wild-type *C. trachomatis* (Yang et al., 2015). The mechanism by which Pgp4 influences exit is unknown, but may involve cooperation with the type III secreted effector CteG (Pereira et al., 2022), a chlamydial protein with unknown function that is expressed independently of Pgp4 (Carlson et al., 2008; Song et al., 2013).

Strikingly, *pgp3* and the PRCLs share a similar expression profile, all are “late genes”, with transcription peaking ~24-28 hours after infection. However, *pgp4* is transcribed across the developmental cycle (Belland et al., 2003). Furthermore, recent work by Zhang et al., has revealed that Euo and Pgp4 co-repress the sigma^66^-dependent promoters of *glgA* and *omcAB* (Zhang et al., 2020) *in vitro*, a finding consistent with a recent study that showed a slight but statistically significant increase in infectious progeny from a recombinant strain when negatively regulating native transcription of *pgp4*via a theophylline-inducible riboswitch (Grieshaber et al., 2022). Thus, Pgp4 mediated regulation is complex, acting as a co-repressor of some late genes but absolutely required for activation of PRCL and Pgp3 expression. A recently published study mapping the sigma^54^ regulon of *C. trachomatis* revealed that conserved PRCLs were directly (CT084, CT142) or indirectly (*glgA*) activated with early extrinsic induction of RpoN and CtcC (Soules et al., 2020b) and this finding was recapitulated when expressed in heterologous *E. coli*, independent of Pgp4. How is Pgp4 modulating expression of this virulence-associated regulon? Zhang et al. suggested Pgp4 interacts with Euo to augment its repressive activity because Pgp4 did not directly bind DNA (Zhang et al., 2020). Alternatively, Pgp4 may engage RNA polymerase directly, or even interact with regulatory factors that contribute to tight control of chlamydial development (Thompson et al., 2015; Soules et al., 2020a; Soules et al., 2020b; Hatch and Ouellette, 2023). In doing so, it may provide *C. trachomatis* with the ability to modulate expression of this virulence regulon in response to altered environmental conditions, as is observed in glucose restricted, infected cells (O’Connell et al., 2011) or in response to heat shock (Huang et al., 2021). Regardless, it is likely that Pgp4-dependent gene regulation is more complex than Pgp4 binding to the promoters of target genes.

It remains to be determined if the role played by Pgp4 in chlamydial infectivity is exclusively through its regulation of Pgp3 and PRCL transcription. Over time, multiple studies have revealed that Pgp3 and PRCLs such as CT049 (Jorgensen and Valdivia, 2008), CT143 (Lei et al., 2021) and even GlgA (Gehre et al., 2016) are secreted into the inclusion or, in the case of Pgp3, to the host cytosol (Lei et al., 2021; Turman et al., 2023). It has been proposed that these proteins are released from chlamydiae via OMVs (Lei et al., 2021). OMVs are made from the outer membranes of Gram-negative bacteria (Schwechheimer and Kuehn, 2015) by an unknown mechanism but there is agreement that OMV production involves one or more of the following three events: the breakdown of links between the outer membrane and peptidoglycan, build-up of cargo proteins in the periplasm leading to bulging of the outer membrane, or rearrangement of lipids on the outer membrane to alter membrane curvature and fluidity (reviewed in (Schwechheimer and Kuehn, 2015)). OMVs are produced by bacteria for a variety of functions such as modulation of host cells and the immune response, acquisition of nutrients, and removal of toxic compounds. The functions of OMVs are largely dependent on the cargo proteins carried within them and their packaging appears to be an actively coordinated process. In *Helicobacter pylori*, the bacterial protease and virulence factor HtrA was enriched in OMVs when compared to the bacterial membrane suggesting it was preferentially packaged for OMV secretion (Olofsson et al., 2010). In this study, adhesins were less abundant in OMVs than the outer membrane providing evidence that bacteria can actively exclude proteins from incorporation into OMVs (Olofsson et al., 2010). Candidate chlamydial OMV cargo proteins have been identified (Frohlich et al., 2012; Frohlich et al., 2014), but no additional mechanistic studies have been published. Our observations (Turman et al., 2023) and those of Lei et al. (Lei et al., 2021) suggest that Pgp4 regulation of OMV secretion extends beyond transcriptional regulation of cargo protein expression, and could involve OMV production or cargo packaging. It seems unlikely that the entirety of the OMV secretion machinery is plasmid-dependent because OMV production and secretion is conserved among many Gram-negative bacteria (reviewed in (Jan, 2017)). Rather, it seems likely that OMV production and secretion in chlamydiae was co-opted by the chlamydial plasmid as a route to virulence effector secretion. This assumption is supported by observations that penicillin and IFN-γ-induced stress result in an accumulation of outer membrane vesicles within the inclusion suggesting chlamydial OMV production may be integrated with a generalized stress response, consistent with published studies for other bacterial species (Mcbroom and Kuehn, 2007; Tashiro et al., 2009; Maredia et al., 2012; McMahon et al., 2012; Macdonald and Kuehn, 2013; Schwechheimer and Kuehn, 2013).

Pgp4 is not the only putative transcriptional regulator encoded on the chlamydial plasmid. Pgp5 was identified as a negative transcriptional regulator after a study of a *pgp5*-deficient strain of *C. muridarum* reported about a three-fold decrease in the transcription of multiple Pgp4-regulated chromosomal loci (Liu et al., 2014a). Interestingly, there was no change in *pgp3* expression when *pgp5* was deleted suggesting that Pgp5 may only regulate a portion of the genes proposed to be regulated by Pgp4 (Liu et al., 2014a). The molecular mechanism underlying Pgp5 regulation of chromosomal loci is unknown. Liu et al. showed that swapping the *C. muridarum pgp5* for its *C. trachomatis* homolog maintained Pgp5-dependent transcriptional regulation (Liu et al., 2014a), which suggests the function of Pgp5 is conserved between *C. muridarum* and *C. trachomatis*. However, transcriptional repression by Pgp5 from *C. muridarum* expressed in either species was more pronounced than repression by Pgp5 from *C. trachomatis*. Further, a previous study characterizing the open reading frames on the plasmid reported no difference in the expression of chromosomal loci in *pgp5*-deficient *C. trachomatis* L2 (Gong et al., 2013). Together, these data could suggest that plasmid-regulated chromosomal loci in *C. muridarum* may be more tightly regulated by Pgp5 than in *C. trachomatis* (Liu et al., 2014a) or that in contrast to the findings of Liu et al, transcriptional regulation by Pgp5 is not conserved between *C. muridarum* and *C. trachomatis*. Studies that include additional serovars of *C. trachomatis* could help clarify the role of Pgp5 as a transcriptional regulator.

A possible role for Pgp5 in disease comes from a single study of *C. muridarum pgp5* deletion and point mutants in the murine genital tract model (Huang et al., 2015). Lower tract shedding from mice infected with *pgp5*-deficient *C. muridarum* was similar to wild-type, but oviduct burdens were lower (Huang et al., 2015). Consequently, only 25-38% of mice infected with *pgp5*-deficient *C. muridarum* developed hydrosalpinx compared to 80% for wild-type *C. muridarum* (Huang et al., 2015). Further, *pgp5*-deficient *C. muridarum* elicited less immune cell infiltration and cytokine secretion in the oviducts of infected mice (Huang et al., 2015).

## 8 Discussion

Ocular and genital infection with *C. trachomatis* can lead to tissue-damaging inflammation. The chlamydial plasmid has been shown to be important for virulence and causing pathology following infection. In the GI tract, the plasmid is associated with increased colonization suggesting the plasmid also plays an important role in maintaining a possible chlamydial GI reservoir in mice.

While data from multiple groups support the role for the plasmid in chlamydial virulence, there has been debate about the extent to which the plasmid mediates virulence. Plasmid-less *C. trachomatis* clinical isolates have been rarely isolated (Peterson et al., 1990; Farencena et al., 1997; Matsumoto et al., 1998; Stothard et al., 1998) suggesting there is active selection for the plasmid. Questions about the extent to which the plasmid mediates virulence remain, particularly regarding effects between and within different areas of a tissue, as well as species-specific differences in the role of the plasmid. The plasmid-associated infectivity defect is most pronounced in the upper genital tract and the small intestine, when compared to the lower genital tract and large intestine, respectively. It is currently unknown what causes these differences. However, it is interesting to note that both the lower genital tract and the large intestine are mucosal sites that are home to a larger, more diverse microflora, when compared to the upper genital tract and the small intestine. This could suggest there are specific interactions between plasmid-encoded or -regulated proteins and processes unique to the small intestine and upper genital tract to promote infectivity. Alternatively, the adaptation of the lower genital tract and large intestine to maintain the microflora may promote a more microbially permissive environment that puts less pressure on less infectious plasmid-deficient chlamydia.

Differences between the role of the plasmid in virulence between tissues has also been observed. Plasmid-deficiency is associated with reduced inflammation in the murine genital tract (O’Connell et al., 2007; O’Connell et al., 2011), suggesting that the chlamydial plasmid is an important driver of tissue-damaging inflammation. In contrast, wild-type plasmid-sufficient *Chlamydia* do not elicit inflammation in the murine GI tract. The basis for these tissue-specific phenotypes is currently unknown. It seems likely that differences in the tissue response to plasmid-dependent chlamydial interactions with host pathways are responsible rather than differences in the specific pathways that *Chlamydia* interact with in each of these tissues. This hypothesis is supported by a study by He et al. that showed lung infection with a plasmid-deficient strain of *C. muridarum* resulted in enhanced infection, inflammation and pulmonary edema, when compared to infection with wild-type *C. muridarum* (He et al., 2011). Increased infection, inflammation and mortality was also observed in TLR2-deficient mice infected with wild-type *C. muridarum* in the lung; evidence for the importance of plasmid-dependent TLR2 activation in controlling lung infection and preventing death (He et al., 2011). Since *C. muridarum* is a natural pulmonary pathogen in mice, maintenance of the plasmid would be evolutionarily important for host survival. In contrast, plasmid-associated TLR2 activation provides no survival advantage when *C. muridarum* is inoculated into the genital tract, but instead, is associated with increased inflammation and tissue damage (O’Connell et al., 2007). Therefore, plasmid-dependent TLR2 activation can lead to tissue-dependent differential outcomes.

These tissue-specific differences may contribute to observed species-specific differences in the relative role of the plasmid in immune pathology. Macaque genital tract infection (Qu et al., 2015) showed no differences in inflammation that were dependent on the plasmid. These observations with *C. trachomatis* are in stark contrast with studies of *C. muridarum* which show it elicits robust cytokine secretion from infected epithelial and immune cells in a plasmid-dependent manner (O’Connell et al., 2007; Frazer et al., 2011). The chlamydial plasmid may contribute more modestly to inflammation during *C. trachomatis* genital tract infection of humans, where infections are mostly asymptomatic and more protracted than in mice. Minor contributions of the chlamydial plasmid to inflammation during human *C. trachomatis* infection would provide an explanation to an evolutionary conundrum surrounding the chlamydial plasmid: why do chlamydiae maintain a plasmid that promotes immune detection and inflammation? As proposed by Russell et al. (Russell et al., 2011), selection for the chlamydial plasmid is likely at the level of infectivity, because maintenance of the plasmid contributes greatly to infectivity, while only contributing modestly to immune detection by human epithelial cells. In the case of *C. muridarum*, a natural murine respiratory pathogen, induction of a robust inflammatory response at the epithelial cell level is likely beneficial. Studies of *Streptococcus pneumoniae* and *Klebsiella pneumoniae* in a murine lung infection model have shown TLR4 activation is associated with increased survival (Branger et al., 2004). Therefore, robust plasmid-mediated TLR2 activation by C. *muridarum* on epithelial and immune cells could have evolved to provide an analogous benefit by limiting infection and severe disease in the lung (He et al., 2011). These observations highlight the need to study the role of plasmid-dependent virulence mechanisms in multiple animal models to gain a full understanding of the role of the plasmid in chlamydial virulence.

Plasmid-mediated virulence to date has been associated with the secreted protein, Pgp3 (Li et al., 2008; Liu et al., 2014b; Ramsey et al., 2014; Hou et al., 2015; Yang et al., 2020; Turman et al., 2023), and the putative transcriptional regulators, Pgp4 (Song et al., 2013; Liu et al., 2014b; Yang et al., 2015; Turman et al., 2023) and Pgp5 (Liu et al., 2014a; Huang et al., 2015). Multiple molecular mechanisms have been proposed for the role of Pgp3 and Pgp4 in mediating infectivity and inflammation. Currently, no single mechanism explains all the observations. None of the proposed mechanisms are mutually exclusive suggesting that the chlamydial plasmid may influence infectivity and inflammation via multiple mechanisms. Further studies examining each of the proposed mechanisms in the context of chlamydial infection with multiple species or serovars could help to determine the relevance of each of the proposed mechanisms during infection. The role of Pgp4 as a transcriptional regulator of chromosomal loci (Song et al., 2013) and its regulation of secretion (Lei et al., 2021; Turman et al., 2023) could result in multiple mechanisms associated with the role for Pgp4 in plasmid-associated virulence. However, it remains unclear how Pgp4 regulates expression of genes on a molecular level and regulates secretion of Pgp3 and other secreted Pgp4-chromosomal loci. Examining the direct roles for Pgp4 in virulence is complex because Pgp4 is required for expression and secretion of multiple genes with unknown functions. Instead, studies of the functions of Pgp4-regulated chromosomal loci could shed light on the connection between Pgp4 and secretion pathways, as well as help further elucidate the role for Pgp4-dependent genes in plasmid-associated phenotypes.

To date, no mechanistic studies have examined the role of Pgp5 in chlamydial virulence. It has been assumed that the role of Pgp5 in virulence is dependent upon its function as a negative transcriptional regulator. However, our current understanding of Pgp4 shows that regulation of virulence in *Chlamydiae* is more complicated. Understanding the role of the plasmid and plasmid-encoded or plasmid-dependent genes in chlamydial virulence will help us understand how chlamydia promote infection and disease at mucosal sites.

## Author contributions

BT wrote the manuscript. CO’C and TD provided content ideas, editing, and feedback on the manuscript. CO’C also made the figure. All authors contributed to the article and approved the submitted version.
